# Genome Sequence of *Lecanicillium fungicola* 150-1, the Causal Agent of Dry Bubble Disease

**DOI:** 10.1128/MRA.00340-19

**Published:** 2019-05-09

**Authors:** Alice M. Banks, Farhana Aminuddin, Katherine Williams, Thomas Batstone, Gary L. A. Barker, Gary D. Foster, Andy M. Bailey

**Affiliations:** a School of Biological Sciences, University of Bristol, Bristol, United Kingdom; Vanderbilt University

## Abstract

The fungus Lecanicillium fungicola causes dry bubble disease in the white button mushroom Agaricus bisporus. Control strategies are limited, as both the host and pathogen are fungi, and there is limited understanding of the interactions in this pathosystem. Here, we present the genome sequence of *Lecanicillium fungicola* strain 150-1.

## Abstract (precis)

The fungus Lecanicillium fungicola causes dry bubble disease in the white button mushroom Agaricus bisporus. Control strategies are limited, as both the host and pathogen are fungi, and there is limited understanding of the interactions in this pathosystem.

## ANNOUNCEMENT

*Lecanicillium fungicola* (Preuss) Zare & Gams [synonym: *Verticillium fungicola* (Preuss) Hassebrauk] (1), an ascomycete fungus of the order Hypocreales, is the causal agent of dry bubble disease of the white button mushroom *Agaricus bisporus*, as well as of other commercially cultivated basidiomycetes (2). Dry bubble disease presents symptoms that include necrotic lesions on mushroom caps, stipe blowout, and undifferentiated tissue masses (2). Some factors involved in this interaction have been proposed based on suppression subtractive hybridization (SSH) and expressed sequence tag (EST) data (3). This disease is of economic importance, causing significant yield/quality losses in the mushroom industry (4). Control methods rely on rigorous hygiene procedures and targeted fungicide treatments; however, increased resistance against these fungicides has been reported (5, 6). Recent taxonomic revisions place L. fungicola close to several arthropod- and nematode-pathogenic fungi rather than to plant-pathogenic Verticillium spp. (1, 7).

*L. fungicola* strain 150-1, a UK isolate of medium virulence obtained from Warwick-HRI, UK (2), was maintained on potato dextrose agar. Genomic DNA was extracted from freeze-dried mycelium using the gentle toluene lysis method of Bonsch et al. (8), and DNA was fragmented to ∼520 bp and sequenced using paired-end 100-base reads with an Illumina HiSeq 2500 instrument, generating 92,464,338 reads. Data were processed using Real-Time Analysis (RTA) 1.17.21.3 with default settings and were demultiplexed with CASAVA 1.8.2. CLC Genomics Workbench 6 (Qiagen Bioinformatics) was used for quality trimming (reads with Phred scores of >20 and reads shorter than 50 nucleotides [nt] were discarded), and an assembly comprising 781 contigs was obtained, spanning 44,574,141 bp and with an *N*_50_ value of 154,124 bp. The GC content was 49.8%.

Contig 84 was identified as the almost-complete mitogenome, and reanalysis of mapped reads to allow circularization of the contig revealed an extra 11 bases required to complete the circular genome, which spans 24,277 bp. The mitogenome shows synteny to that of the nematophagous fungus Lecanicillium saksenae (9), including identical gene order and just one intron in *rn1*, again reflecting the close phylogenetic relationship between these fungi. Manual annotation was performed by comparison to closely related mitogenomes (9, 10) with tRNAs identified using tRNAscan-SE (11).

Some of the symptoms of dry bubble, such as cap spotting, might be due to secondary metabolites. Analysis using fungiSMASH 5.0.0 (12) revealed the following 38 biosynthetic gene clusters for secondary metabolites: 8 polyketide synthases (PKSs), 21 nonribosomal peptide synthase (NRPS) or NRPS-like clusters, 3 PKS-NRPS hybrids, 5 terpene synthases, and 1 indole cluster. Analysis of these gene clusters is ongoing.

The interaction between *L. fungicola* and its primary host, *A. bisporus*, remains far from fully characterized. While the disruption of β-1,6 glucanase resulted in reduced virulence (13), knockout of the *pmk1*-like mitogen-activated protein (MAP) kinase did not affect virulence (14), indicating that the models established for fungal plant pathogens might not readily apply to fungus-fungus interactions.

The genome sequence of *L. fungicola* provides a useful basis to uncover the molecular mechanisms underlying pathogenicity in the *L. fungicola-A. bisporus* interaction.

### Data availability.

This project has been deposited in DDBJ/ENA/GenBank under the accession number FWCC00000000 and BioProject number PRJEB19844. The genome assembly version is the first version and includes accession numbers FWCC01000001 to FWCC01000781. Raw sequencing data have been deposited under accession number ERR3181828. The annotated mitogenome has been deposited under accession number LR536627.
